# Overexpression of Potato *PYL16* Gene in Tobacco Enhances the Transgenic Plant Tolerance to Drought Stress

**DOI:** 10.3390/ijms25168644

**Published:** 2024-08-08

**Authors:** Panfeng Yao, Chunli Zhang, Zhenzhen Bi, Yuhui Liu, Zhen Liu, Jia Wei, Xinglong Su, Jiangping Bai, Junmei Cui, Chao Sun

**Affiliations:** 1 State Key Laboratory of Aridland Crop Science, Gansu Agricultural University, Lanzhou 730070, China; yaopf@gsau.edu.cn (P.Y.); zhangchunl@st.gsau.edu.cn (C.Z.); bizz@gsau.edu.cn (Z.B.); lyhui@gsau.edu.cn (Y.L.); liuzhen@gsau.edu.cn (Z.L.); wej@st.gsau.edu.cn (J.W.); suxl@st.gsau.edu.cn (X.S.); baijp@gsau.edu.cn (J.B.); cuijm@gsau.edu.cn (J.C.); 2 College of Agronomy, Gansu Agricultural University, Lanzhou 730070, China

**Keywords:** potato, drought tolerance, StPYL16, expression analysis

## Abstract

PYR/PYL/RCAR proteins are abscisic acid (ABA) receptors that play a crucial role in plant responses to abiotic stresses. However, there have been no research reports on potato PYL so far. In this study, a potato *PYL* gene named *StPYL16* was identified based on transcriptome data under drought stress. Molecular characteristics analysis revealed that the StPYL16 protein possesses an extremely conserved PYL family domain. The tissue expression results indicated that the *StPYL16* is predominantly expressed at high levels in the underground parts, particularly in tubers. Abiotic stress response showed that *StPYL16* has a significant response to drought treatment. Further research on the promoter showed that drought stress could enhance the activation activity of the *StPYL16* promoter on the reporter gene. Then, transient and stable expression of *StPYL16* in tobacco enhanced the drought resistance of transgenic plants, resulting in improved plant height, stem thickness, and root development. In addition, compared with wild-type plants, *StPYL16* transgenic tobacco exhibited lower malondialdehyde (MDA) content, higher proline accumulation, and stronger superoxide dismutase (SOD), peroxidase (POD), and catalase (CAT) activities. Meanwhile, *StPYL16* also up-regulated the expression levels of stress-related genes (*NtSOD*, *NtCAT*, *NtPOD*, *NtRD29A*, *NtLEA5*, and *NtP5CS*) in transgenic plants under drought treatment. These findings indicated that the *StPYL16* gene plays a positive regulatory role in potato responses to drought stress.

## 1. Introduction

Plant growth and development are frequently compromised by a variety of abiotic stresses, including cold, drought, and salinity. Abiotic stress is a major hidden danger in agricultural production, commonly resulting in reduced crop yields [1]. Following exposure to abiotic stress, plants need to orchestrate physiological and biochemical responses, along with gene regulation, to acclimate to adverse environmental conditions [2]. Gene regulation is intricately linked to stress-induced hormonal signaling pathways. Studies have demonstrated that the plant hormone ABA plays a pivotal role in mediating plants’ responses to abiotic stress [3].

The ABA signal is initially recognized and triggered by the presence of ABA, with ABA perception achieved through the binding of ABA receptors to the hormone [4]. The predominant type of ABA receptors is the pyrabactin resistance (PYR)/PYR-like (PYL)/regulatory component of the ABA receptor (RCAR) complex, which localizes to the nucleus and cytosol [4]. The PYR/PYL/RCAR (referred to hereafter as PYL) ABA receptor, in conjunction with protein phosphatase 2C (PP2C) and SNF1-related protein kinase 2 (SnRK2), forms the core ABA signaling network, which features a dual negative regulatory mechanism [4,5]. In the absence of ABA, PP2Cs bind to SnRK2s, thereby suppressing the activity of SnRK2 proteins. Upon an elevation in ABA levels, ABA is recognized and bound by its receptors, initiating interaction between the ABA receptor complex and PP2Cs. Consequently, SnRK2s are released from inhibition, leading to the activation of downstream target gene expression [5,6]. In plants, numerous ABA receptors, protein phosphatases, and kinases have been recognized as pivotal elements in the ABA signaling pathway. For instance, *Arabidopsis* possesses 14 PYR/PYL receptors, 76 PP2C phosphatases, and 10 SnRK2 kinases [7,8]. Following research on Arabidopsis, members of the PYL, PP2C, and SnRK2 families have also been identified in other plants such as rice [9], maize [10,11], and tomato [12].

As a stress hormone, the concentration of ABA in plants increases rapidly under abiotic stress conditions, particularly drought and salt stress [5,13]. In recent years, the role of ABA receptors in plant responses to abiotic stress has been gradually elucidated. Overexpression of *AtPYL4/RCAR10*, *AtPYL5/RCAR8*, and *AtPYL13/RCAR10* enhanced the drought resistance of transgenic Arabidopsis [14,15]. Meanwhile, *AtPYL9/RCAR1* enhanced drought resistance through the promotion of leaf senescence, thereby reducing transpiration and facilitating water transport to young tissues [15]. Furthermore, ABA receptors AtPYL1/RCAR12 and AtPYL3/RCAR13 in *Arabidopsis* have been identified to play crucial roles in extreme temperature responses, with their overexpression in plants demonstrating significant tolerance to cold and heat stress [16]. The functional role of *PYL* genes has been observed in a variety of food and commercial crops. For example, overexpression of certain PYL genes, such as *OsPYL3*, *9*, and *10*, enhances drought and cold tolerance in transgenic rice [17,18]. Similarly, overexpression of *OsPYL5* improves drought and salt tolerance in rice [19]. In wheat, overexpression of *TaPYL4* enhances grain production under drought conditions and increases water-use efficiency [20]. PYL family genes have also been identified in fruit crops like grape [21], sweet orange [22], banana [23], and apple [24]. However, research on the biological functions and regulatory roles of these genes in the stress responses of potatoes is limited. 

The potato (*Solanum tuberosum* L.) plays a vital role in global food security as the fourth largest crop worldwide. However, it often encounters various stresses, especially drought, during growth in regions like Gansu, China, known for low precipitation [25]. Our team focuses on developing drought-resistant potato varieties, identifying drought-resistant genes, and analyzing regulatory mechanisms. Progress has been achieved in studying the hormone regulation of potato growth and stress responses. However, the characterization of potato *PYL* genes and their functions in drought stress responses remain largely unexplored. Based on transcriptome data from drought-stressed potatoes, we identified a significant *PYL* gene responsive to drought stress and designated it as *StPYL.* Through scrutinizing the reaction of the *StPYL16* gene and its promoter to drought stress, the drought resistance capacity of *StPYL16* was subsequently validated through overexpression in tobacco. This pioneering research delves into the role of the *PYL* genes within potatoes, showcasing pivotal implications in unraveling the ABA signaling pathway in potatoes amidst drought challenges.

## 2. Results

### 2.1. Screening and Molecular Characterization Analysis of StPYL16

**Figure 1:**
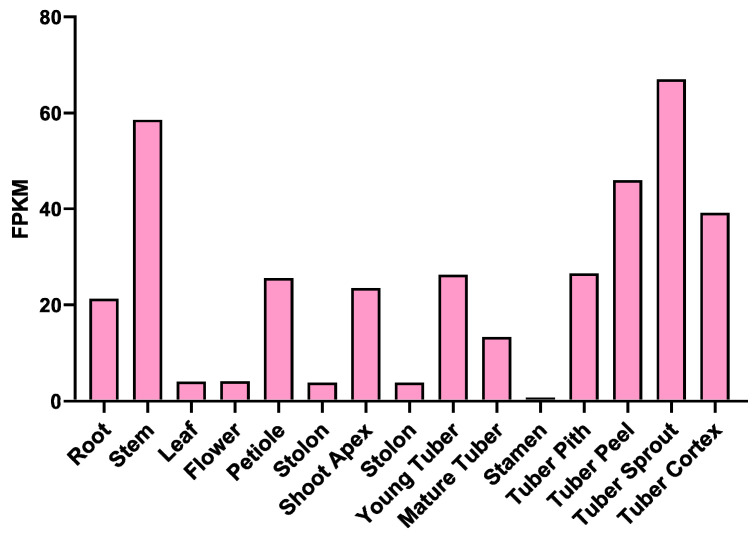
Tissue-specific expression of *StPYL16* gene. Data from Spud DB Potato Genomics Resource website (http://spuddb.uga.edu/, accessed on 23 May 2024).

PYL serves as a crucial ABA receptor in plant resistance against abiotic stresses, such as drought. Consequently, our team conducted a thorough analysis of the potato *PYL* gene family at an early stage, identifying a total of 23 potato *PYL* gene sequences (Figure S1). Subsequently, we examined the expression profiles of all *PYL* genes in two potato varieties with varying levels of drought resistance using transcriptome data. This analysis aimed to pinpoint the key *PYL* genes that play a role in the potato’s response to drought stress. Ultimately, we identified the gene PGSC0003DMT400003773, which exhibited a significant response to drought stress, and designated it as *StPYL16* (Figure S2). Tissue-specific expression analysis revealed that *StPYL16* is predominantly expressed in the below-ground organs, encompassing the young tuber, mature tuber, tuber pith, tuber peel, tuber sprout, and tuber cortex. Conversely, in the above-ground organs, its expression is notably elevated in the stem while relatively subdued in other tissues (Figure 1).

### 2.2. StPYL16 Is Strongly Induced by Drought Treatment

**Figure 2:**
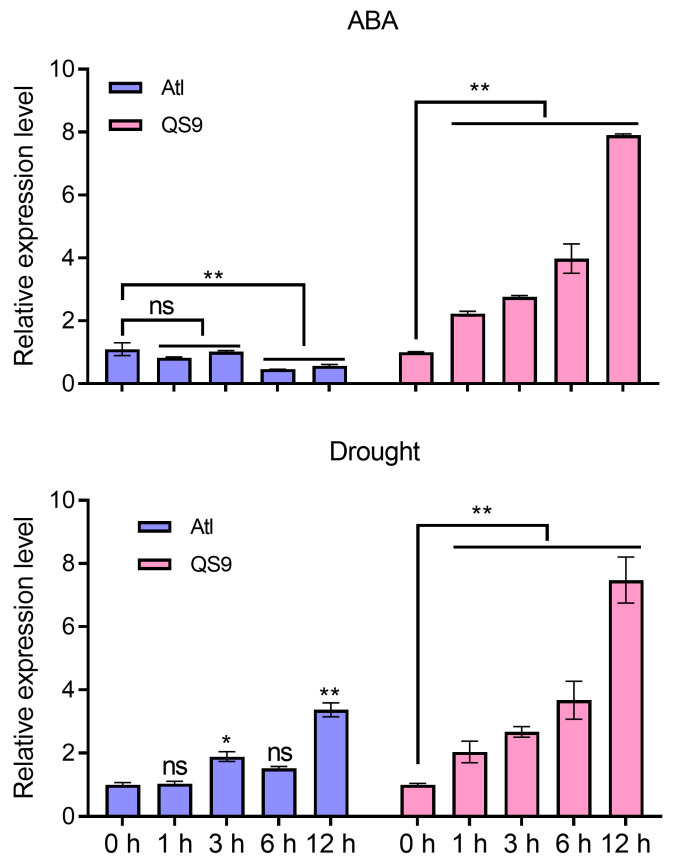
The relative expression level of the *StPYL16* gene under 100 µM ABA and 200 mM mannitol stress treatments. ‘Atl’ and ‘QS9’ represent drought-sensitive and drought-tolerant potato varieties, respectively. For the ABA treatment, 20-day-old potato seedlings were subjected to spraying with 100 M ABA, while other 20-day-old seedlings were treated with ethanol as a control. For the drought treatment, 20-day-old potato seedlings were transferred to a liquid MS medium containing 200 mM mannitol, with seedlings under normal liquid MS conditions serving as the control. Above-ground samples were collected for gene expression analysis at 0, 1, 3, 6, and 12 h post stress induction under both treatment conditions. Data represent the means ± SD of three replicates. * and ** indicate significant difference at *p* < 0.05 and *p* < 0.01 levels, respectively. ns indicates that the difference is not significant.

In order to clarify the response of *StPYL16* to abiotic stress, its expression levels under ABA and drought treatment were further analyzed by qRT-PCR (Figure 2). Two varieties, Atl (drought sensitive) and Q9 (drought tolerant), were chosen for the study. Under ABA treatment conditions, *StPYL16* exhibited a pronounced response in QS. Significant differences were observed after 1 h of stress, and its expression level gradually increased as the treatment duration extended, peaking at 12 h. At this point, the expression level was 7.98 times higher than that at 0 h. In contrast, In drought-sensitive materials, *StPYL16* exhibited an inverse pattern, showing a significant decrease after 6 h of stress followed by stabilization. Under drought stress, *StPYL16* demonstrated a similar expression pattern to that under ABA treatment in QS9, with a notable increase in expression from the onset of stress, reaching its peak after 12 h. In the Atl, there were significant differences in *StPYL16* expression levels at 3 and 12 h of stress, but the overall expression level did not show a regular trend.

### 2.3. Drought Enhances the Activity of StPYL16 Promoter

**Figure 3:**
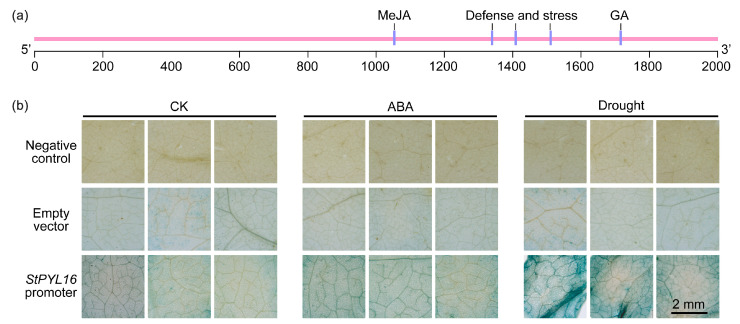
Stress response analysis of *StPYL16* gene promoter. (**a**) Analysis of stress response elements in *StPYL16* gene promoter. (**b**) GUS histological staining analysis of transient transformation of tobacco with *StPYL16* gene promoter under 100 µM ABA and 200 mM mannitol treatment.

In order to further elucidate the mechanism by which *StPYL16* responds to stressful environments, a 2000 bp promoter fragment upstream of the *StPYL16* gene start codon was obtained using TBtools. PlantCARE (http://bioinformatics.psb.ugent.be/webtools/plantcare/html/, accessed on 25 March 2024) was employed for predicting the regulatory elements within the promoter region. The findings showed that the identified *cis*-elements fall into two main categories according to their responsive functions: hormone-responsive elements and stress-responsive elements (Figure 3a). Furthermore, the promoter sequence also contains various other types of regulatory elements, such as photoresponsive elements and developmental-related elements.

To examine the response of the *StPYL16* promoter to ABA and drought stress, the *proStPYL16* sequence was isolated from potato genomic DNA and inserted into the pBI101–GUS vector for further analysis. Under normal conditions, transgenic plants containing *proStPYL16* displayed a blue coloration in histological GUS assays, while the negative control exhibited a faint yellow color (Figure 3b). This observation suggested that *proStPYL16* activates the downstream GUS reporter gene expression, indicating its intrinsic promoter strength. Subsequent exposure to drought stress resulted in a more intense GUS staining pattern in transgenic tobacco plants, suggesting an enhancement in the promoter activity of *proStPYL16* under drought conditions. Conversely, no significant difference in GUS staining was observed between ABA treatment and normal conditions, implying that the *proStPYL16* promoter may not be responsive to ABA. These findings collectively suggested that *StPYL16* may function as a drought-responsive gene.

### 2.4. Transient Transformation of StPYL16 Improves Drought Tolerance of Transgenic Tobacco

**Figure 4:**
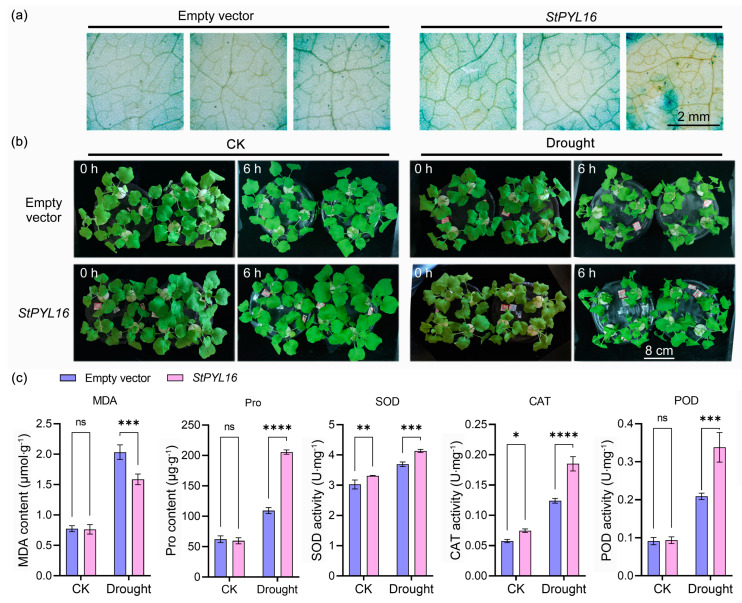
Identification of drought resistance in tobacco after transient transformation of *StPYL16* gene. (**a**) GUS histochemical staining of *StPYL16* transgenic plants; (**b**) phenotypic collection of each genotype before and after drought stress; (**c**) determination of physiological indexes related to stress. *, **, ***, and **** indicate significant difference at *p* < 0.05, *p* < 0.01, *p* < 0.001, and *p* < 0.0001 levels, respectively. ns indicates that the difference is not significant.

To preliminarily investigate the function of *StPYL16*, we transiently expressed it in tobacco and subjected it to drought treatment to explore whether it can affect the drought resistance of transgenic tobacco. Initially, GUS staining results of the transgenic plants revealed that leaves transformed with empty vector and *StPYL16* exhibited a distinct blue color, indicating successful introduction of the target gene (Figure 4a). Concurrently, overexpression of this gene resulted in a certain degree of improvement in the wilting degree of plants compared to the control, indicating that the expression of *StPYL16* enhanced the drought resistance of tobacco (Figure 4b). 

Moreover, to elucidate the impact of *StPYL16* on transgenic plants, we assessed the levels of MDA and Pro, which are closely linked to plant stress resistance, as well as the activity of antioxidant enzymes (SOD, POD, and CAT). The findings demonstrated that over-expression of *StPYL16* led to a substantial increase in Pro accumulation in transgenic plants under drought stress while decreasing the MDA content. Conversely, under normal conditions, no significant disparity was observed between the two indicators. The activity of antioxidant enzymes displayed a varied pattern. SOD and CAT activities were significantly higher under both control and stress conditions. POD activity exhibited a notable increase under drought stress, with no significant difference observed between transgenic and control plants under normal conditions (Figure 4c).

### 2.5. Generation of Stable Transgenic Tobacco

**Figure 5:**
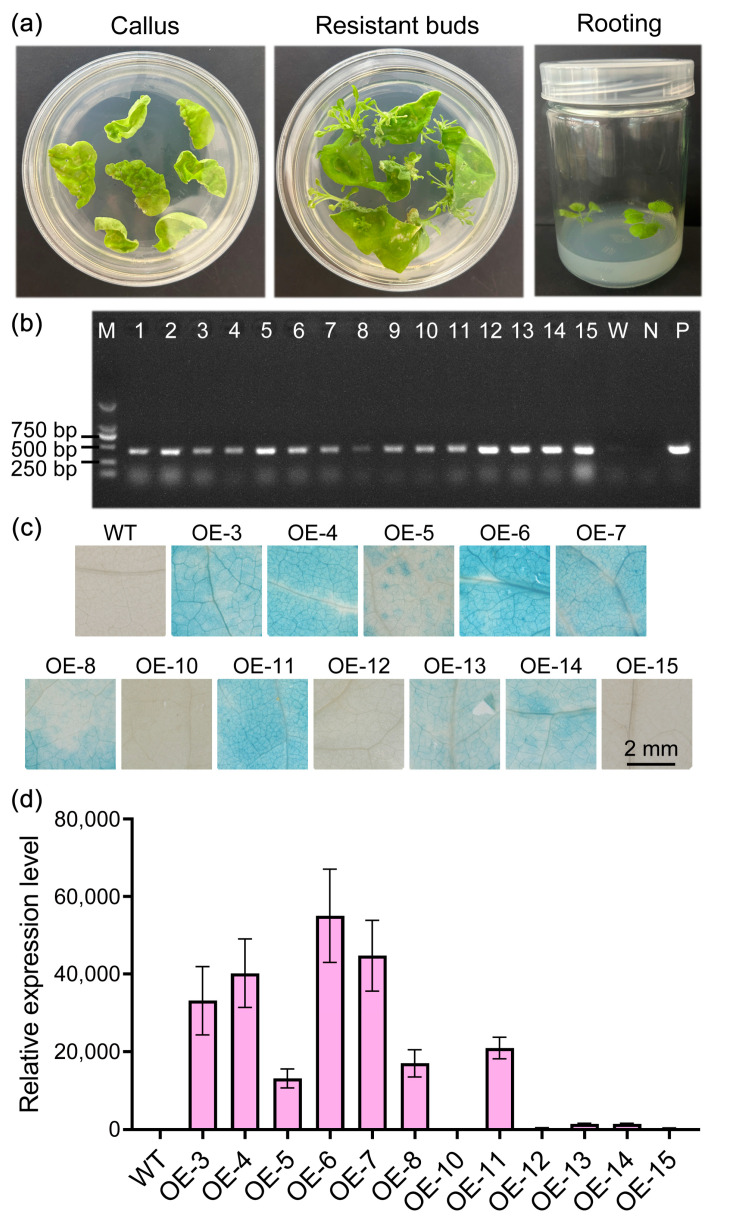
Positive identification of *StPYL16* transgenic tobacco. (**a**) The process of transforming *StPYL16* into tobacco; (**b**) PCR molecular identification of positive *StPYL16* transgenic lines; (**c**) GUS histochemical staining of *StPYL16* transgenic lines and control plants; (**d**) analysis of expression level of *StPYL16* in transgenic lines.

To further elucidate the function of *StPYL16* under drought stress conditions, transgenic tobacco plants overexpressing *StPYL16* were generated using the *Agrobacterium*-mediated leaf disk transformation method (Figure 5a). Fifteen transgenic plants with resistance were selected through kanamycin screening and qRT-PCR analysis (Figure 5b). The results revealed that *StPYL16* was expressed in all resistant plants but not in the wild-type control plants. Consistent results were obtained from GUS histochemical staining (Figure 5c). Subsequently, we quantified the expression levels of *StPYL16* in each positive plant using qRT-PCR and identified three transgenic lines (OE-4, OE-6, and OE-7) with high levels of *StPYL16* expression for further investigation (Figure 5d).

### 2.6. Overexpression of StPYL16 Increases Tobacco Drought Tolerance

**Figure 6:**
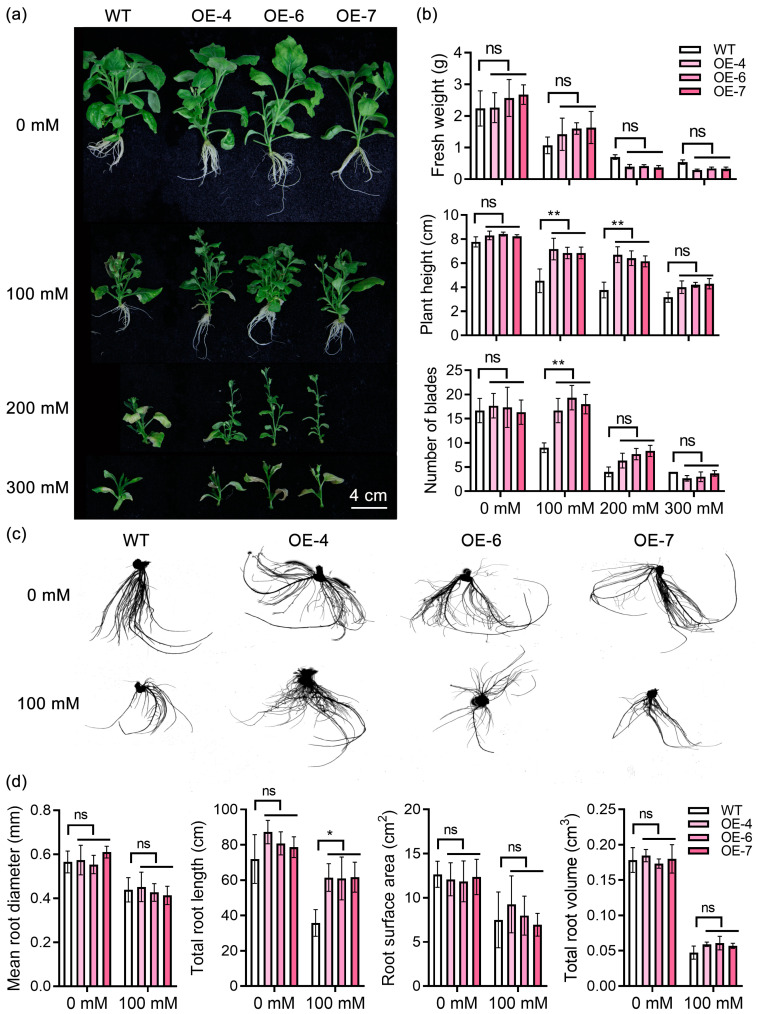
Drought-resistance function of *StPYL16* after stable transformation of tobacco. Transgenic tobacco stem segments with consistent growth were transferred to MS medium as well as MS medium supplemented with 100 mM, 200 mM, and 300 mM mannitol for 30 days to stress, followed by the measurement of various phenotypic traits. Additionally, root traits of the plants were analyzed using a root scanner (LD-WinRHIZO). (**a**) Phenotypic identification of plants under drought stress; (**b**) statistics of phenotypic indicators of plants under drought stress; (**c**) root scanning diagram of each plant under different treatment conditions; (**d**) determination of root system related indexes of various plants under different treatment conditions. * and ** indicate significant differences at *p* < 0.05 and *p* < 0.01 levels, respectively. ns indicates that the difference is not significant.

To investigate the phenotypic changes of transgenic tobacco before and after drought stress, photographs were taken of transgenic lines and wild-type plants under various treatments for observation. The findings indicated that under normal conditions, there was no notable distinction between each transgenic strain and the control plant. However, under stress conditions, the growth of all plants was impeded, with the control plants exhibiting a more remarkable inhibition. Specifically, at 100 mM, the plant height and root development of transgenic plants were significantly superior to those of the control. Conversely, at 200 mM and 300 mM, the root growth of all plants was completely suppressed (Figure 6a).

Further measurements were conducted on a variety of phenotypic indicators. The results revealed that the over-expression of *StPYL16* did not impact the fresh weight of transgenic plants under drought conditions. Plant height, as a crucial indicator reflecting plant growth and development, was significantly higher in *StPYL16* transgenic plants compared to control plants under 100 and 200 mM conditions. The height of the three transgenic lines nearly reached 1.5–1.8 times that of the control. Similarly, the number of leaves displayed a comparable pattern, with transgenic plants under 100 mM stress exhibiting significantly more leaves than the control, reaching 2.1 times the quantity (Figure 6b).

**Figure 7:**
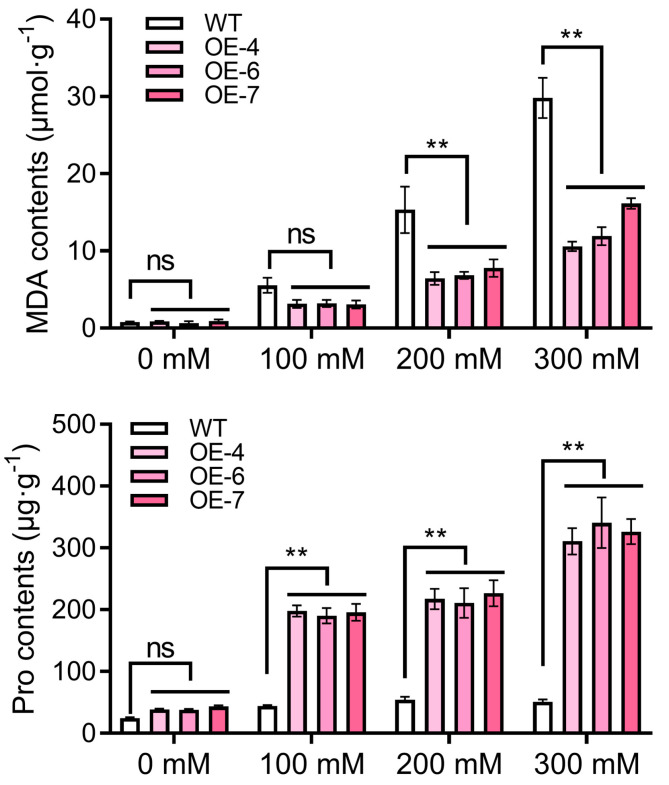
Determination of malondialdehyde (MDA) and proline (Pro) contents in different plants under different treatment conditions. Transgenic tobacco stem segments with consistent growth were transferred to MS medium as well as MS medium supplemented with 100 mM, 200 mM, and 300 mM mannitol. After 30 days of stress treatment, aboveground tissues were selected for the measurement of physiological indices. ** indicates significant difference at the *p* < 0.01 level. ns indicates that the difference is not significant.

Furthermore, phenotype scans and related data indicator measurements were performed on the root systems of each plant (Figure 6c). The findings indicated that the root length of *StPYL16* transgenic plants was notably greater than that of the control under drought stress. While other indicators such as root diameter, root surface area, and root volume showed some variances, these distinctions were not statistically significant (Figure 6d). Finally, two key physiological indicators closely associated with drought stress, MDA and Pro, were measured (Figure 7). Under normal conditions, no significant differences were observed between the lines. However, under drought conditions, the MDA content in each transgenic line was significantly lower than that in the control, whereas the Pro content was notably higher than that in the control. This disparity became more pronounced as the stress level intensified. In conclusion, the over-expression of *StPYL16* significantly bolstered the drought resistance of transgenic tobacco.

### 2.7. Stress-Related Gene Expression in Transgenic Tobacco Plants under Drought Stress Mediated by StPYL16

**Figure 8:**
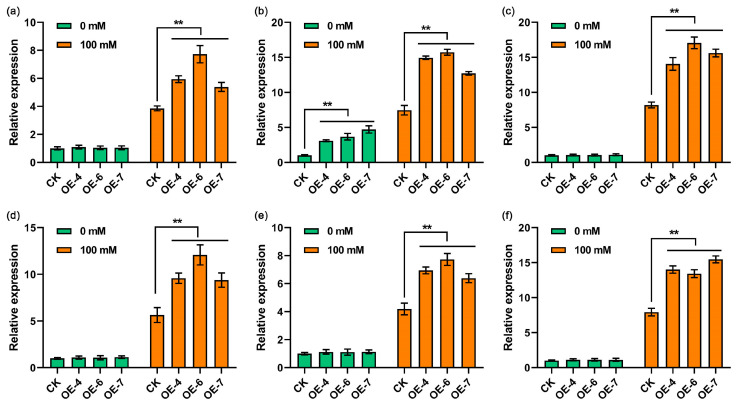
Expression analysis of stress-related genes in transgenic plants and wild-type plants. Transgenic tobacco stem segments exhibiting consistent growth were transferred to MS medium and MS medium supplemented with 100 mM mannitol. Following a 30-day stress treatment period, aboveground tissues were collected for gene expression analysis. (**a**–**f**) represent *NtSOD*, *NtCAT*, *NtPOD*, *NtRD29A*, *NtLEA5*, and *NtP5CS* genes, respectively. ** represents significant differences between transgenic lines and WT at *p* < 0.01. ns indicates that the difference is not significant.

To investigate the mechanism behind the enhanced drought tolerance in *StPYL16* transgenic plants, we performed a comparative analysis of the expression patterns of stress-related genes in *StPYL16* transgenic lines and control plants under both normal and drought conditions. Upon exposure to drought stress, certain stress-related genes, including *NtSOD*, *NtCAT*, *NtPOD*, *NtRD29A*, *NtLEA5*, and *NtP5CS*, exhibited significantly elevated expression levels in *StPYL16* transgenic plants when compared to control plants (Figure 8). Nevertheless, there was no notable disparity in gene expression levels, except for the *NtCAT* gene, between *StPYL16* transgenic plants and control plants that were grown under normal conditions. Notably, following drought stress, the expression levels of all examined genes in both wild-type and transgenic plants showed a marked increase compared to normal conditions (Figure 8). These findings suggested that the over-expression of *StPYL16* may modulate the expression of these stress-related genes to bolster plant resilience against drought stress.

## 3. Discussion 

The hormone ABA plays a crucial role in regulating plant growth, development, and stress responses [26,27]. Drought stress often leads to increased ABA synthesis, with the PYL protein playing a key role in mediating plant drought resistance through ABA signaling pathways [28]. Although the function of the *PYL* gene in improving plant drought tolerance has been thoroughly examined across a range of plant species, there is a lack of detailed functional studies on the specific role of *PYL* in potatoes.. This current research endeavor focuses on the cloning of *StPYL16*, a gene belonging to the PYL family, from potato plants. Subsequently, we conducted analyses to elucidate the regulatory role of *StPYL16* in conferring drought resistance in transgenic tobacco plants following transient and stable expression.

With the continuous advancement of sequencing technology, an increasing number of plant genomes have been sequenced, facilitating the identification of gene families crucial for plant growth and stress response regulation within the entire genome. Despite the significant role of ABA in this process, it was not until 2009 that PYL was recognized as an ABA receptor through research involving the utilization of a synthetic ABA agonist known as pyranose [8]. Subsequent to this discovery, numerous studies have focused on the screening and identification of PYL family proteins across various plant species. In Arabidopsis thaliana, 14 PYL proteins were delineated into three subfamilies based on phylogenetic analysis as well as structural and functional characterization [29]. A genome-wide examination of plant PYL family proteins revealed variations in the number of PYL family genes among different plant species. For example, there are 15 in tomato [30], 14 in poplar [31], 40 in *Gossypium hirsutum* [32], 29 in tobacco [31], 8 in grape [21], 13 in rice [33], and 38 in wheat [34]. Despite the significant differences in the quantity of PLY family genes, the clustering pattern of these genes remains consistent across plant species. PYL family members in these plant species are classified into three subfamilies. In the current study, 23 *PYL* genes were identified in potato, a number similar to the 29 found in tobacco, possibly due to their classification within the Solanaceae family. Additionally, phylogenetic analysis demonstrated that potato PYL proteins were categorized into four subgroups, a slight deviation from previous reports suggesting most plant PYL proteins are divided into three subgroups. This difference may be associated with the complex chromosome structure of potatoes or the emergence of new genes with structural variances during evolution.

Drought, as a prominent abiotic stress factor, presents a formidable challenge to worldwide agricultural production, resulting in substantial annual decreases in crop yields. Plant hormones, notably ABA, play a pivotal role in plant responses to abiotic stress and the modulation of resistance mechanisms [26,27]. The PYL protein, serving as an ABA receptor, plays a vital part in ABA perception and signal transduction. Numerous studies have recognized its role in enhancing plant drought tolerance. For instance, the overexpression of *AtPYR1*, *AtPYL1*, *AtPYL2*, *AtPYL3*, *AtPYL8*, and *AtPYL9* has been shown to significantly enhance the drought tolerance of transgenic Arabidopsis [15,35]. Similar results have been observed in other plant species, such as rice [36], corn [37], wheat [20], poplar [38], and cotton [39]. Through transcriptome analysis under drought stress in the early stages, we identified a member of the PYL gene, *StPYL16*, that may participate in the response to drought stress. Subsequent investigations involving promoter stress response detection and transgenic functional validation demonstrated that *StPYL16* exerts a positive regulatory effect on potato response to drought stress (Figure 1). Similar to several PYL genes in previous studies, the overexpression of *StPYL16* mitigates damage from drought stress, resulting in improved growth conditions in transgenic plants. Furthermore, the increased activity of antioxidant enzymes suggests that *StPYL16* enhances plant drought tolerance by facilitating the removal of excess reactive oxygen species under stress conditions. Comparable outcomes have been reported in studies focusing on gene families in other plant species [40]. Further research is warranted to uncover the mechanisms through which PYL enhances antioxidant enzyme activity under stress conditions. Meanwhile, we examined the effect of ABA on *StPYL.* We found that the *StPYL16* showed no significant response to exogenous ABA in two different drought-resistant varieties. Thus, our results suggested that *StPYL16* are involved in plant stress resistance through an ABA-independent pathway.

When plants are subjected to drought stress, there is a disruption in the balance between intracellular activity production and clearance, leading to the accumulation of reactive oxygen species, which in turn causes oxidative damage and plant cell death. This phenomenon has been extensively studied and documented [41,42]. In the face of drought stress, plants elevate the transcription levels of antioxidant enzyme genes such as *NtGPX*, *NtSOD*, *NtPOD*, and *NtCAT*, leading to a subsequent escalation in the activity of SOD/CAT/POD enzymes [43,44]. These changes play a crucial role in enhancing plant drought resistance. In our study, it was found that overexpression of StPYL16 significantly boosted the expression of *SOD/CAT/POD* genes and the corresponding antioxidant enzyme activity in transgenic plants under drought stress conditions. Significantly, under standard growth conditions, the expression level of the *CAT* gene in StPYL16-overexpressing plants exhibited a marked increase compared to that in control plants. This observation was corroborated by the reduction in MDA content in transgenic plants (Figure 4), indicating reduced oxidative damage. The reactive oxygen species scavenging system is vital for plants to counteract drought stress, involving both enzymatic (such as superoxide dismutase, catalase, peroxidase, and GPX) and non-enzymatic scavengers. These enzymes work synergistically to mitigate the harmful effects of reactive oxygen species [44,45]. MDA, a byproduct of lipid oxidation, serves as a marker for plant oxidative damage [46]. Proline is an osmolyte found in plant cytoplasm that plays a crucial role in stabilizing membrane and protein conformation, clearing reactive oxygen species, and reducing photodamage to chloroplast thylakoid membranes [47]. In the current study, overexpression of *StPYL16* led to an increase in free proline content in transgenic plants under drought stress conditions, corresponding to the upregulation of the proline synthesis gene *NtP5CS* (Figure 4). Proline biosynthesis involves the glutamate and ornithine pathways, utilizing glutamate and ornithine as precursors to catalyze the production of pyrroline-5-carboxylate (P5C) by P5CS and OAT enzymes, respectively [48]. The accumulation of proline in plants under abiotic stress conditions is typically linked to the upregulation of P5CS and OAT expression, along with the downregulation of proline dehydrogenase (ProDH) and pyrroline-5-carboxylate dehydrogenase (P5CDH) expression [49]. Hence, the findings suggested a positive correlation between the increased Pro levels in transgenic plants under drought stress and the enhanced expression of *NtP5CS*. 

## 4. Materials and Methods

### 4.1. Plant Material and Growth Conditions

The potato varieties “Atlantic” and “Qingshu 9” along with tobacco “*Nicotiana tabacum* (NT12)” were stored at the State Key Laboratory of Aridland Crop Science, Gansu Agricultural University. Potato seedlings were cultivated in an artificial growth chamber with conditions of 16 h/8 h light/darkness, 25 °C, and 60% relative air humidity. Tobacco seeds were planted in a greenhouse with a temperature of 25 ± 2 °C, with a photoperiod of 12 h/12 h light/darkness and a relative humidity of 60%. The plant expression vectors pCAMBIA1304 and *Agrobacterium tumefaciens* GV3101 were kept in our laboratory.

### 4.2. Isolation and Characterization of StPYL16

Total RNA was extracted from the samples using an RNAout kit (Tiandz, Beijing, China) and then reverse transcribed into cDNA with the RevertAid First Strand cDNA Synthesis kit (MBI, Woburn, MA, USA). Based on the transcriptome data of potato seedlings under drought stress, a novel *PYL* gene named *StPYL16* was identified using specific primers. The gene sequence was analyzed using the NCBI database. Detailed information on the primers used can be found in Supplementary Table S1.

### 4.3. Expression Analysis of StPYL16

The tissue-specific expression of *StPYL16* in potato and its reactions to various abiotic stresses were detected using qRT-PCR. Twenty days post-planting, potato seedlings were sprayed with 100 µM ABA or alcohol (as a control). For drought treatment, 20-day-old seedlings cultivated in untreated medium were shifted to liquid MS medium supplemented with 200 mM mannitol, with samples collected at various time intervals (0, 1, 3, 6, and 12 h) for gene expression analysis. Seedlings moved to normal MS medium were used as the reference. Each stress treatment was repeated three times for precision. The qRT-PCR primer details can be found in Supplementary Table S1.

### 4.4. Promoter Cis-eElement Analysis

The 2000 bp promoter sequences upstream of the start codon of *StPYL16* were extracted using TBtools. Subsequently, the sequence was analyzed for *cis*-acting elements using the PlantCARE website (http://bioinformatics.psb.ugent.be/webtools/plantcare/html/, accessed on 23 May 2024). Next, the *StPYL16* promoter was inserted into the plant expression vector pBI101-GUS through homologous recombination. Tobacco leaves were transiently transformed with *Agrobacterium* tumefaciens GV3101, and GUS staining was conducted after 2 days to assess promoter activity. Meanwhile, tobacco plants with the *StPYL16* promoter were exposed to stress by transferring them to Hoagland solution containing 200 mM mannitol, with control plants growing in normal Hoagland solution. Additionally, hormone treatment involved spraying with 100 mM ABA, while another group was sprayed with alcohol as a control. After 6 h of treatment, samples were collected for GUS histochemical staining.

### 4.5. Evaluation of Drought Resistance after Transient Transformation of Tobacco

The coding sequence (CDS) of *StPYL16* was obtained through PCR amplification using specific primers (Table S1). The PCR product was then digested with *Bgl* Ⅱ and *Spe* Ⅰ before being inserted into the pCAMBIA1304 vector to generate the pC1304-StPYL16 construct. This construct was subsequently introduced into *Agrobacterium* tumefaciens GV3101 using the heat shock method for further experiments. To investigate the function of *StPYL16*, tobacco plants were transferred from normal growth conditions to Hoagland nutrient solution after approximately 20 d of growth and cultured for 2 d. Following this, tobacco leaves were infiltrated with GV3101 carrying pC1304-StPYL. The empty vector pC1304 was used as a negative control. After 2 d of normal growth, both the experimental and control samples were exposed to stress by transferring the entire plants into Hoagland nutrient solution supplemented with 200 mM mannitol. Samples were collected after 6 h for GUS staining to confirm successful transformation. Additionally, various physiological parameters related to stress response were also evaluated.

### 4.6. Evaluation of Drought Resistance after Stable Transformation of Tobacco

The recombinant plasmid pC1304-StPYL16 was used for transformation of *Nicotiana tabacum* (T12) via the *Agrobacterium*-mediated leaf disk method. The transgenic plants were then cultured on Murashige and Skoog (MS) medium and screened with 1/2 MS medium containing kanamycin (50 mg L^−1^, *w*/*v*) for selection. Positive lines were identified and confirmed through qRT-PCR and GUS staining. From the qRT-PCR results, three transgenic lines showing the highest expression of *StPYL16* were selected. These lines were then cut into uniform stem segments and subjected to stress by inoculation on MS medium supplemented with 100 mM, 200 mM, and 300 mM mannitol, while normal MS medium was used as the control. After a 30-day stress period, the physiological indicators related to stress were evaluated.

### 4.7. β-Glucuronidase (GUS) Staining

Histochemical staining for GUS activity was performed as described previously [50]. The transgenic plant leaves were harvested and immersed in the reaction solution (2 mM 5-bro-mo-4-chloro-3-indolyl-β-d-glucuronic-acid, 50 mM sodium phosphate, 10 mM EDTA, 2 mM ferricyanide, and 0.1% Triton X-100, pH 7.0) and incubated at 37 °C for 12 h under dark conditions. Subsequently, the stained leaves underwent gradient elution with 30%, 60%, and 100% ethanol in an 80 °C water bath. Following the complete disappearance of chlorophyll, photomicrographs were captured using an optical microscope.

### 4.8. Determination of Phenotypic and Physiological Indicators

The proline and MDA contents were determined according to a previous publication [51]. The enzymatic activities of superoxide dismutase (SOD), peroxidase (POD), and catalase (CAT) in both the transgenic lines and control plants were measured using commercial assay kits (BC0170, BC0220, and BC0200; Beijing Solarbio Science & Technology Co., Ltd., Beijing, China) with spectrophotometric methods. Phenotypic traits primarily consisted of plant height, stem diameter, and root length. Additionally, a root scanner (LD-WinRHIZO) was employed to assess parameters such as total root length, number of root tips, root area, and root volume of the plant root system.

### 4.9. Expression Measurement of Stress-rResponsive Genes

Total RNA was extracted from a tobacco plant and reverse transcribed to synthesize cDNA. The expression of stress-related genes was assessed by qRT-PCR using specific primers (Table S1).

### 4.10. Statistical Analysis

Three plants were sampled for each of the three biological replicates in every treatment. Mean and standard deviation (SD) were calculated for each treatment based on data collected from these replicates. The variance between the three overexpression (OE) lines and control plants was evaluated using one-way analysis of variance (ANOVA) in SPSS v19.0 (SPSS Inc., Chicago, IL, USA), with statistical significance set at *p* < 0.05 or *p* < 0.01.

## 5. Conclusions

In conclusion, this research unveiled the significance of the potato ABA receptor gene *StPYL16* in the plant’s response to drought stress. Moreover, the over-expression of *StPYL16* has been shown to boost plant performance under drought conditions. Significantly, our study offers a valuable approach for genetically modifying future crops to adapt to progressively adverse environments.

## Data Availability

All data are available within the manuscript.

## Supplementary Materials

The following supporting information can be downloaded at: https://www.mdpi.com/article/10.3390/ijms25168644/s1.
