# Supplementary material for: Overexpression of Potato PYL16 Gene in Tobacco Enhances the Transgenic Plant Tolerance to Drought Stress
Source: Int J Mol Sci. 2024 Aug 8;25(16):8644. doi: 10.3390/ijms25168644 (PMC11354512; doi:10.3390/ijms25168644)
Supplement: Supplementary file 1 [file ijms-25-08644-s001.zip › Suplementary figures.pdf]

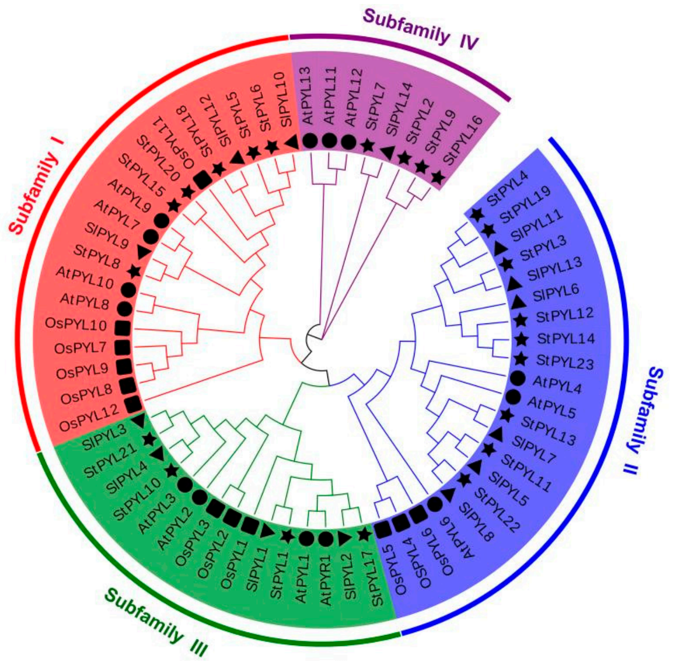

Figure S1. Unrooted phylogenetic tree representing the relationships among *StPYL* genes from *Solanum tuberosum* and *PYL* genes from other species. The phylogenetic trees were constructed using the neighbor-joining (NJ) method in MEGA7.0 and the default parameter value was set to 1000.

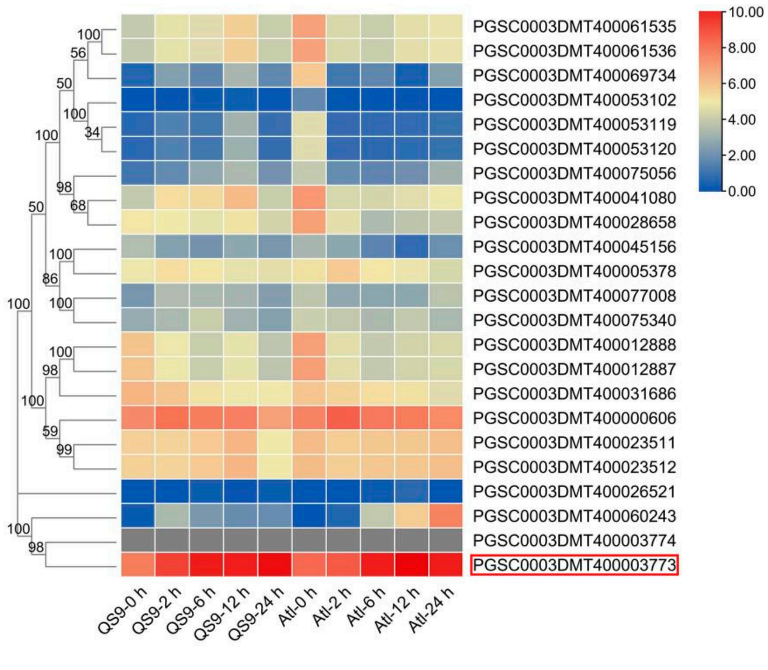

Figure S2. Heatmap showing expression of *StPYL* genes based on RNA-seq data. Heatmap was generated based on log2 FPKM. ‘QS9’ and ‘Atl’ represent drought tolerant potato variety ‘Qingshu 9’ and drought sensitive potato variety ‘Atlantic’, respectively. Three-week-old potato seedlings were treated in 1/2 MS medium with final concentration of 200 mM mannitol, and the transcriptome was sequenced after 0, 2, 6, 12 and 24 h of stress. The red box represents *StPYL16*.
