# Supplementary material for: Overexpression of Potato PYL16 Gene in Tobacco Enhances the Transgenic Plant Tolerance to Drought Stress
Source: Int J Mol Sci. 2024 Aug 8;25(16):8644. doi: 10.3390/ijms25168644 (PMC11354512; doi:10.3390/ijms25168644)
Supplement: Supplementary file 1 [file ijms-25-08644-s001.zip › Suplementary tables.pdf]

Table S1 The sequences of primers were used in the study.

Gene	Sequences	Function
StPYL16	F: 5'- ATGGGTGTGAATACCTATACTTGTGA-3'	Gene clone
	R: 5'- TTAAGCGTAGACAGAAGGATTCG-3'	
StPYL16	F: 5'-GTGAGTCAACGACCACAATTTCCC-3'	qRT-PCR
	R: 5'-TGCTTCCAACACCACCATCTCC-3′	
NtRD29A	F: 5'-TCGGTGTACCAACAGGCATA-3'	
	R: 5'-CCCTTGCTTTGGTGTTGTTT-3'	
NtP5CS	F: 5'-TGGCCCTCCCCGTAATCCAGATTC-3'	
	R: 5'-GATACATTCCCCATGTAGCACTT-3'	
NtNtLEA5	F: 5'- TTGTTAGCAGGCGTGGGTAT-3'	
	R: 5'- CTCTCGCTCTTGTTGGGTTC-3'	
NtPOD	F: 5'- GCTGTTCGACGAGTTGTTAA-3'	
	R: 5'- CTCTGGCTGAGTTGTTGTTGG-3'	
NtSOD	F: 5'-AGCTACATGACGCCATTTCC-3'	
	R: 5'- CCCTGTAAAGCAGCACCTTC-3'	
NtCAT	F: 5'- AAGAATGGCTCCGCAAGTTA-3'	
	R: 5'- GCCTAGCAATTCCAGAGTGG-3'	
NtActin	F: 5'-CAAGGAAATCACCGCTTTGG-3'	
	R: 5'-AAGGGATGCGAGGATGGA-3'	
